# Government Protection Needed

**Published:** 1883-01

**Authors:** Henry B. Baker


GOVERNMENT PROTECTION NEEDED. By HENRY B. BAKER.

Permit me to ask your attention and the attention of the public and its representatives in congress to the urgent need for legislation immediately on the assembling of congress, which shall make an appropriation for the continuance of the immigrant inspection service, now partially sustained by the National Board of Health. By reductions in force and in pay that board has been able to continue the service until Dec. 15, but after that time it will have to stop, unless Congress will immediately pass a bill or joint resolution which shall provide especially for the continuance of the immigrant inspection service. Experience would seem to suggest that it would be wise to intrust the disbursement of the funds and the supervision of the service to the National Board of Health in preference of any other United States department or service.

During the 18 weeks ending Sept. 30, 1882, there were inspected at Port Huron about 18,000 immigrants, and at Detroit about 23,000—nearly 41,000 immigrants at the two points. Of these who passed through Port Huron a large number had not been previously inspected; over 12,000 certificates of protection from small-pox were issued at that port, nearly 8,000 immigrants having been vaccinated there during the 18 weeks, and thus that number protected from getting and spreading small-pox. At the two stations 178 sick in transit were found, 85 of whom had measles. This brief summary may convey an idea of the extent of the labor in this State; but the necessity for such a service may be apparent by referring to the incessant struggle which the health authorities throughout this and other States have had and are having with small-pox and other contagious diseases which, since the immigration has been so great, have been introduced at so many places and so frequently that it seems impossible much longer to prevent these diseases from becoming generally epidemic throughout this country, more particularly the northern and western States where the immigrant and other travel is great. Thus, in Detroit a special meeting of the board of health was recently called to consider the question of closing the schools because of the prevalence of diphtheria. A late paper says: "Public funerals are forbidden in Boston Mass., on account of the prevalence of diphtheria and other contagious diseases." In Cincinnati cases of small-pox have been so numerous, it is alleged, that the health officials have suppressed the facts in order to prevent a panic; but the number reported has been large.

Michigan has hardly been free from small-pox in the past year, and at many places, such as Grand Rapids and Detroit, the local health authorities have been almost constantly engaged in combatting it until some time after the inspection service began. Diphtheria and scarlet fever have been constantly present in many parts of the State; and they break out at new places about as fast as they are controlled at others. Some of the contagious diseases are yet constantly being introduced, notwithstanding the government inspection, because the service has been mainly confined to preventing the introduction and spread of small-pox. The deaths from each of several of the other contagious diseases greatly outnumber those from small-pox. These several facts show the importance of not only continuing the immigrant inspection service with respect to small-pox, but also granting the protection of the U. S. government from the continuous introduction from abroad of other contagious diseases, especially diphtheria and scarlet fever. It is believed that neither small-pox nor other communicable disease are originated in Michigan, but are so continually being brought into this country and into the State by means of commerce and immigration, that neither the local or the State boards of health are able to free the country or the State from these contagious diseases. The attempt is even more difficult than would be the case with the several fire departments if articles capable of spontaneous combustion were being thus frequently and generally introduced.

If local health authorities undertake to stop the introduction of disease, their best methods will cause delay to travel and interference with what seems to be of national concern. The expense, also, of the inspection, at Port Huron, for instance, is too great to be borne by that city; especially as the danger from the introduction of diseases at Port Huron extends to every part of the northwest, as far as Manitoba. A large part of the small-pox in Michigan has been traced to Chicago; but without immigrant inspection Chicago gets contagious diseases by way of Port Huron, as well as by other routes. Port Huron is a port where great numbers of immigrants first touch United States soil, and is a point where it is of great importance that there should be a thorough inspection and many primary vaccinations. Detroit is a point where it seems very desirable there should be an inspection station for inland inspection and surveillance; a few immigrants also first enter this country at that port.

Before the inspection service began, there were frequent importations of small-pox into this country and this State by immigrants; but since this service has been established its influence extends backward on the lines of travel, and is effective for good on board the ship, on which the immigrant sails, and it continues to his destination and the necessary surveillance after his arrival at his destination; consequently the importations of small-pox have been very much less in number, because the knowledge that rigid inspection is enforced in Michigan and Illinois, etc., tends to make the examinations and vaccinations on board ship and at ports of entry more complete and thorough, and the local health authorities can be informed of the entrance of immigrants into their jurisdictions, and the public health be in some measure protected.

What is needed is that the immigrant inspection be continued and the inspectors directed to pay more attention than heretofore to scarlet fever and diphtheria and to the disinfection of all luggage believed to be infected with any disease especially dangerous to the public health. At the present time no person should take the risks of travel without the protection of vaccination. The vaccination of unprotected immigrants is needed not only for their own protection because unusually exposed to small-pox en route, but also to prevent them from spreading the diease broadcast throughout this country.—*Lansing Republican.*

